# Gender-specific improvements in cognitive resources: Impact of a multidimensional exercise program on healthy physically inactive older adults

**DOI:** 10.1007/s00391-024-02405-1

**Published:** 2025-01-31

**Authors:** Marlene Krumpolt, David Rahil, Anneke Schumacher, Lucas Sannemann, Kerstin Witte

**Affiliations:** Institut III, Sportwissenschaft, Otto-von Guericke-Universität Magdeburg, Zschokkestr. 32, 39104 Magdeburg, Germany

**Keywords:** Executive functions, Aging, Reaction speed, Selective attention, Exekutive Funktionen, Altern, Reaktionszeit, Selektive Aufmerksamkeit

## Abstract

**Background:**

Neuroanatomical parameters deteriorate with age and this process varies among individuals. Gender differences in these parameters have been documented but their effects on cognition remain unclear. Physical activity, continuous learning and social interactions are recognized strategies to prevent cognitive decline.

**Aim:**

This study investigated the effects of multidimensional training on selective attention and reaction speed in physically inactive but healthy older adults, exploring gender-specific differences in cognitive abilities.

**Material and methods:**

The study employed a pre-post design and included a 24-week exercise program. A total of 60 participants (30 male, 30 female) aged 65–69 years completed the program, which consisted of 90 min of fitness and 90 min of recreational sports each week. Cognitive performance was assessed using the STROOP (a visual test for selective attention) and reaction time (RT) tests administered through the Vienna Test System.

**Results:**

Significant gender differences were observed. Women were initially slower than men but significantly improved their reaction speed after the training (*p* < 0.001, d = 1.144). Conversely, men showed significant improvements in controlled and automated actions (*p* = 0.021, d = 0.5).

**Discussion:**

Multidimensional training enhances cognitive performance in physically inactive older adults. Gender-specific differences in reaction time were confirmed, while differences in other cognitive domains were revealed. The underlying causes of these differences are still unclear, raising the question of whether training programs should be tailored differently for men and women.

## Abstract (de)

**Hintergrund:**

Mit zunehmendem Alter verschlechtern sich neuroanatomische Parameter individuell. Geschlechtsspezifische Unterschiede sind belegt, ihre Auswirkungen auf die Kognition sind jedoch unklar. Zur Vorbeugung des kognitiven Abbaus gehören körperliche Aktivität, kontinuierliches Lernen und soziale Interaktionen.

**Ziel:**

In dieser Studie werden die Auswirkungen eines multidimensionalen Trainings auf die selektive Aufmerksamkeit und die Reaktionsgeschwindigkeit bei körperlich inaktiven, aber gesunden Senioren untersucht, wobei der Einfluss des Geschlechts berücksichtigt wird.

**Material und Methoden:**

Die Studie wurde in einem Prä-Post-Design durchgeführt und umfasste ein 24-wöchiges Sportprogramm. Sechzig Teilnehmer (30 Männer, 30 Frauen) im Alter von 65 bis 69 Jahren absolvierten das Programm, das wöchentlich 90 min Fitness- und 90 min Freizeitsport mit einer Reihe von Breitensportarten umfasste. Die kognitiven Leistungen wurden mit Hilfe des STROOP- und RT-Tests über das Wiener Testsystem bewertet.

**Ergebnisse:**

Es wurden signifikante geschlechtsspezifische Unterschiede festgestellt. Frauen waren langsamer als Männer, verbesserten aber ihre Reaktionsgeschwindigkeit nach dem Training signifikant (*p* < 0,001; d = 1,144). Männer verbesserten signifikant kontrollierte und automatisierte Handlungen (*p* = 0,021; d = 0,5).

**Diskussion:**

Multidimensionales Training verbessert die kognitiven Leistungen sportlich inaktiver älterer Erwachsener. Geschlechtsspezifische Unterschiede in der Reaktionszeit wurden bestätigt, während Unterschiede in anderen exekutiven Funktionen aufgedeckt wurden. Die Ursachen sind noch unklar, und ob Männer und Frauen unterschiedlich trainieren sollten.

Research on cognitive aging is crucial as the global population ages. A key challenge is preserving cognitive health in old age. Although aging affects individuals differently, a general decline in brain structure occurs in later adulthood. While gender differences in brain development exist, their effect on cognition remains unclear. Several factors can prevent cognitive decline and support mental performance in old age, with physical activity playing a significant role. It helps prevent cognitive impairment, enhancing attention and responsiveness.

The dominant image of aging is characterized by ideas about the loss of physical and mental functions but these changes are always heterogeneous [18]. In particular, an inactive lifestyle in middle and old age leads to a loss of performance [17]. Physical activity, continuous learning and social interactions are crucial for optimal cognitive health in old age [3]. Changes in the brain during aging are reflected in mental performance, with a decrease in gray matter, especially in the prefrontal area, being associated with impaired memory, attention and concentration, particularly executive functions [21]. Numerous interventions aim to maintain or improve the cognitive performance of older adults [13]. Dancing, in particular, has a significant impact on cognitive function as it activates brain regions through continuous cognitive and motor learning [1]; however, dancing does not appeal to everyone and men, in particular, are more likely to choose sports involving competition [15]. Above all, a varied sports program also appeals to previously inactive but healthy men [24]. Generally, previously inactive but healthy seniors have fewer opportunities to gain the benefits of each sport as there are few suitable offers for newcomers or returnees. Additionally, the research on training and its effect on healthy, inactive seniors remains unclear.

## Background

Executive functions, i.e., the ability to control attention, are most affected by advancing age. These skills heavily depend on activity in the frontal cortex. The prefrontal cortex supports focusing attention, processing complex information and planning. As people age the volume of the brain decreases. The brain’s degenerative process typically begins around ages 30–40 years. Cognitive abilities decline further after the age of 60 years [14]. Age-related cognitive decline is linked to structural brain changes and synapse loss but the molecular mechanisms remain unclear. Some regions, like the prefrontal lobe, shrink earlier and faster. The “last in, first out” theory suggests that the brain’s most recently developed parts deteriorate first [6]. Accident analyses also indicate that a significant number of registered traffic accidents in older adults are due to attentional errors and reduced responsiveness [26].

As brain plasticity decreases, older adults require good cognitive stimulation, new challenges, opportunities to learn new things, social interaction and physical activity [7]. This study examines the following research questions: to what extent does multidimensional training influence selective attention and reaction speed in previously inactive but healthy older adults? Furthermore, are there gender-specific differences in cognitive abilities?

### Reaction speed

Reaction speed is the ability to react as quickly and precisely as possible to one or more environmental stimuli. It can be understood within the context of the CHC model (Cattell-Horn-Carroll model) of human cognitive abilities, a well-established and validated theoretical framework [19]. Reaction speed is measured as the time between the occurrence of a stimulus (processing reaction) and an observable reaction (motor response) [9]. In the domain of general speed, secondary factors such as processing speed are considered. At the primary level, distinctions can be made in reaction and decision speed, including simple reactions and choice reactions.

Simple reactions respond to a single stimulus, while choice reactions involve selecting from multiple alternatives. Reaction time is measured using a reaction test (RT), where participants press a button for relevant stimuli and then return to the rest button. Studies show that reaction time declines with age [25].

### Selective attention

Selective attention is suppressing dominant information to respond to new and non-dominant information. Athletes are also better at filtering irrelevant environmental stimuli compared to novices [10]. The prefrontal cortex, especially the dorsolateral prefrontal cortex, is vital for directing and controlling attention. Selective attention involves automated and conscious/controlled actions. Differences in milliseconds between baseline and interference conditions indicate the tendency for interference. A smaller difference suggests effective control in both automatic and conscious actions. Practiced actions become automatic, whereas unfamiliar actions demand more attention [3]. Conscious actions occur in the prefrontal cortex, while learned actions are shifted to deeper regions such as the basal ganglia. Older adults have fewer cognitive resources but as actions become more automated, the brain can work more effectively [20].

## Methods

### Study design

The study was conducted in pre-post design, with a 24-week sports program as an intervention. Cognitive tests for executive functions were conducted at two measurement points: pre-test at baseline (t0) and post-test after 24 weeks (t1). The research protocol adhered to the principles of the Declaration of Helsinki and received approval from the Ethics Commission of Otto von Guericke University Magdeburg, Germany (No. 3/22).

### Sample

The study included 30 men and 30 women aged between 65 and 69 years (mean 67 ± 1.4 years). Recruitment of participants was conducted through newspaper advertisements. All participants were physically and mentally unimpaired but had not done any regular sporting activity for at least 2 years. Before enrolment, the standardized MMSE (Mini-Mental State Examination) (mean 28 ± 1.2) was administered to rule out cognitive impairments.

### Intervention

A total of 60 participants completed a 6-month exercise program, which included weekly 90-min fitness sessions and 90 min of recreational sports. The fitness sessions focused on improving coordination, strength, flexibility, and endurance, while the recreational sessions introduced activities like dancing, racket sports and ball games, teaching basic techniques and playful exercises. Designed for inactive seniors, the program started with low intensity and gradually increased. It also included practical and theoretical education on healthy nutrition. The interdisciplinary approach combined diverse activities to challenge participants and promote social interaction, benefiting group dynamics and mental attitudes.

### Data recording and statistical analyses

Cognitive functions were assessed using the Vienna test system (Wiener Test System, WTS) [23]. Reaction speed was evaluated using RTS1 (simple reaction) and RTS4 (choice reaction), while the STROOP test assessed (visual) selective attention [2, 27]. Both tests provided insights into executive function and cognitive control. Motor reaction times in milliseconds (motor response) refer to the physical responses initiated by cognitive processing, involving the activation and coordination of muscles to perform voluntary or involuntary movements. Processing reactions in milliseconds (processing time) encompass the mental activities that interpret sensory information and generate appropriate responses. Both reactions were recorded during the tests.

The STROOP task involved four conditions: two baselines for reading and color naming, and two interference conditions with incongruent words and colors, one focused on reading the word and the other on naming the color. The time difference in milliseconds between these conditions and the baselines indicated interference propensity, with reading as an automated action and naming as a controlled action. Paired t‑tests assessed pre-post comparisons, and the Mann-Whitney U test was used to compare genders at each measurement point. Normal distribution was checked with the Kolmogorov-Smirnov test. Effect sizes were calculated using Cohen’s d or Pearson’s r. Data were analyzed with SPSS (Version 29.0).

## Results

### Reaction time

**Fig. 1:**
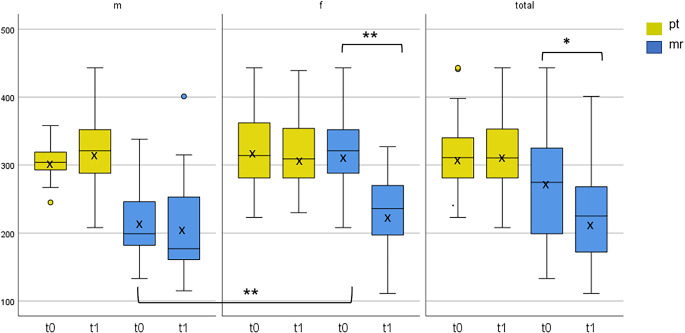
Processing time and motor response in ms (Y-axis). Processing time (pt) and motor response (mr) in ms of men (*n* = 30) and women (*n* = 30) and as a total (*n* = 60) of baseline (t0) and post-test (t1) during the single reaction condition (RTS1). ** *p* < 0.001 (highly significant), * *p* < 0.05 (statistically significant)

First, a comparison was made between men (m) and women (f) at t0 and t1 and over the entire study period. A significant difference (*p* < 0.001; d = 1.77) was found between m and f at t0 (m = 219.29 ± 57.60 ms; f = 319.62 ± 55.72 ms) for motor responsiveness (mr) during the single reaction condition (RTS1) (Fig. 1). At t1, women were still slower but not with significant difference compared to the men (m = 209.38 ± 72.19 ms; f = 232.90 ± 57.47 ms) (Fig. 1).

**Fig. 2:**
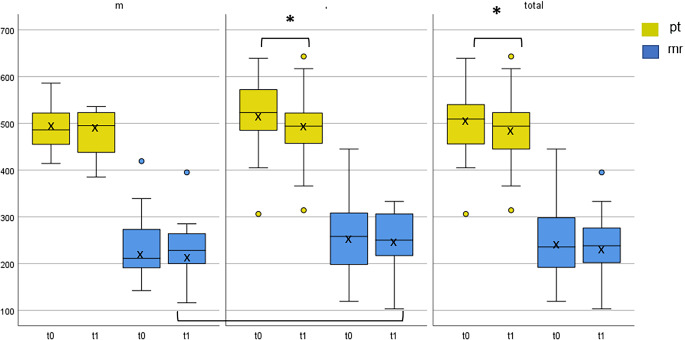
Processing time and motor response*. *Time (pt) and motor response (mr) in ms (Y-axis) of men (*n* = 30) and women (*n* = 30) and as a total (*n* = 60) of baseline (t0) and post-test (t1), during the selection response condition (RTS4). * *p* < 0.05 (statistically significant)

Considering mr during the RTS4, it was observed that m were also faster; however, a trend (*p* = 0.073; d = 0.33) was only evident at t1 (m = 227.14 ± 61.28 ms; f = 251.00 ± 64.95 ms) (Fig. 2). The ability of processing time (pt) during RTS1 is noteworthy. Although not significant, m became slower from t0 to t1 compared to f (m = 305.19 ± 27.51 ms; 319.62 ± 12.15 ms; f = 323.86 ± 62.0 ms; 314.57 ± 11.75 ms). During the RTS4, both m and f became faster in pt but there was no significant difference between the sexes. Only females showed a time effect from t0 to t1 regarding mr during RTS1 and pt during RTS4, with significant improvements (mr: *p* < 0.001, d = 1.144; pt: p = 0.006, d = 0.602) (Figs. 1 and 2).

Examining the complete sample size, significant improvements in pt during RTS4 and mr during RTS1 (pt: 269.45 ± 75.57 ms/221.14 ± 65.54 ms; *p* = 0.009, r = 0.382; mr: 269.45 ± 75.57 ms/221.14 ± 65.54 ms; *p* = 0.001, r = 0.63) from t0 to t1 were observed, although these values were strongly influenced by f (Figs. 1 and 2). On the other hand, looking at the results of pt during RTS1, one also recognizes a not significant deterioration over time (314 ± 7.45 ms/317 ± 8.36 ms), which can be attributed to the drop in performance in effect for men (Fig. 1).

### Selection attention

**Fig. 3:**
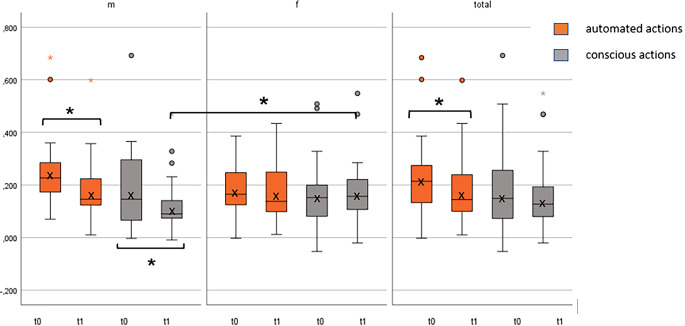
Selection attention in ms (Y-axis). Selective attention of men (m), *n* = 30 and women (f), *n* = 30, and of total (*n* = 60) from baseline (t0) and post-test (t1) during STROOP reading (automated action) and naming interference condition (conscious action). * *p* < 0.05 (statistically significant)

The results of selective attention also reveal differences between the sexes. The results of the women did not show a significant improvement in any condition. Men were significantly better in the controlled action during t1 (m = 0.131 ± 0.11 ms; f = 0.035; *p* = 0.0035; r = 0.33) and the time course of the automated action (t1 = 0.248 ± 0.15 ms; t2 = 0.185 ± 0.12 ms) presented a significant improvement with a strong effect (*p* = 0.021, d = 0.5). There was also a trend from t0 to t1 with a significance of *p* = 0.05; r = 0.042, indicating that men improved in the controlled action (t1 = 0.184 ± 0.16 m; t2 = 0.1131 ± 0.11 ms) (Fig. 3). Looking at the data as a whole, a different picture emerges. There was no significant change over time in the controlled action. A significant improvement with a medium effect was observed in the automated action (0.219 ± 0.13 ms/0.182 ± 0.12 ms; *p* = 0.015; r = 0.37) (Fig. 3).

In connection with the gender-specific division, it is clear that the results were strongly influenced by men. Men were faster than women in performing the STROOP task but completed the tasks with more errors, which can only be seen at measurement time t0. There was no difference between men and women at t1. A comparison of t0 with t1 shows that men significantly improved in both the automated action and the controlled action and made fewer errors (*p* = 0.005, r = 0.436/p = 0.042, r = 0.44). The values for women did not significantly change (Table 1).

**Table 1:** Number of errors during STROOP reading interference (automated action) and naming interference (conscious action) from baseline (t0) to post-test (t1)

			*t0*	t1		
Outcomes	Variable	*n*	*M* *±* *SD*	M ± SD	*p*	r
Automated action (number of errors)	Male	30	7.38 ± 22.36	1.19 ± 1.83	0.005	0.436
	Female	30	1.9 ± 1.64	1.05 ± 1.41	0.063	0.287
	Total	60	4.64 ± 15.9	1.12 ± 1.62	0.001	0.51
Conscious action (number of errors)	Male	30	6.67 ± 25.96	0.95 ± 1.143	0.042	0.44
	Female	30	2.05 ± 2.45	1.38 ± 2.22	0.265	0.24
	Total	60	4.40 ± 18.37	1.17 ± 1.86	0.034	0.33

*M* mean value, *SD* standard deviation, *p* significance < 0.05, *r* effect size

The data for the total group show a significant improvement in the number of errors during automated action (*p* = 0.001) as well as a significant time effect during conscious action (*p* =0.034) (Table 1).

## Discussion

The results of these studies show how important the gender-specific subdivision of cognitive abilities in people aged 64–69 years is, as the ’total’ results are often influenced by one gender. One study found that loss of white matter integrity is a major cause of age-related cognitive decline in cognitively normal older adults [12]. Solving cognitive tasks is partially linked to and influenced by the brain’s anatomical structure. Some brain regions such as the hippocampus and hypothalamus are particularly affected by age-related changes in men, whereas the anterior cingulate cortex shows greater vulnerability in women. That could be a reason for gender-specific differences in cognitive abilities in aging [28]. Executive functions, especially those linked to the prefrontal cortex, play a role in decision-making about relevant and irrelevant stimuli, as seen in tasks like the STROOP. Studies indicate that, on average, men have faster reaction times than women, a difference that persists into older age [4]. This is consistent with our findings; however, women tend to be slower than men but achieve a greater intervention effect during RT. Particularly during STROOP tasks, differences could be more related to the slower reaction times observed.

Improvement in selective attention through physical activity interventions aligns with studies describing a similar effect of exercise on executive function [16]; however, the type of intervention influences the effect. Ingold et al. (2021) described differences between open (dynamic, unpredictable, e.g., tennis) and closed (predictable, standardized, e.g., swimming) skills, with open activities significantly impacting inhibitory abilities [11]. This diverse sports program offers both open and closed activities, thereby maximizing the benefits of both activities. A study like that of Langhammer et al. suggests that healthy older adults ideally engage in at least 150 min of exercise per week for a minimum of 6 months [16]. This is consistent with our program. Moreover, research indicates that moderate-intensity training is associated with improved working memory and cognitive flexibility, while high-intensity training enhances inhibition [5]. These findings align with evidence that 6 months of multidimensional training positively influences cognitive resources. Intensive training impacts selective attention more than aerobic training in older adults.

Gender-specific differences in intervention outcomes may stem from variations in brain structure that influence cognition. Women might need distinct incentives, potentially favoring aerobic activities. Physiological differences such as hormone disparities, especially the decline in estrogen postmenopause, can impact cognitive functions and reaction times. Research indicates that hormone therapies may improve cognitive performance in postmenopausal women, highlighting a hormonal component to these gender differences. [22]. Menopause, in particular, exhibits long-lasting effects. Many women experience “brain fog” referring to cognitive symptoms such as memory difficulties and attention issues; hormones play a significant role in cognition [8].

The results indicate that regular and varied physical activity positively influences cognitive performance in older adults, especially inhibition and processing speed. Further studies are needed to understand gender-specific training modalities. Imaging techniques are being utilized to further understand functional brain activity and sex differences, based on these studies.

### Limitations

An enlargement of the sample size is necessary to enhance the study’s validity. Additionally, gender differences in symptoms such as migraine or poor sleep during measurement periods may contribute to varied outcomes between men and women; however, these factors were not assessed in the current research. The individual needs of the different genders should be investigated in other age groups. Furthermore, it is essential to evaluate different training approaches for both genders.

## Practical conclusion

- Cognitive enhancement: sedentary older adults (64–69 years) can improve cognitive abilities through multidimensional training.
- The study highlights gender differences in cognitive abilities
- The training program had varying effects by gender, underlining the need for more gender-specific research.
- The diverse training approach benefits both genders but showed significant improvements, especially in men.
- Further research is needed on gender differences in cognitive enhancement and to develop targeted programs for inactive older adults
- Gender-specific differences in cognitive abilities should also be examined in other age groups of older adults

## Data Availability

The data sets and protocols from this study are available upon request from the corresponding authors.
